# Enhanced Uncoupling of the Mitochondrial Respiratory Chain as a Potential Source for Amyotrophic Lateral Sclerosis

**DOI:** 10.3389/fneur.2013.00086

**Published:** 2013-07-03

**Authors:** Michael Hoffmann

**Affiliations:** ^1^Department of General Pediatrics, University Children's Hospital, Heinrich Heine UniversityDusseldorf, Germany

Amyotrophic lateral sclerosis (ALS) is a fatal neurodegenerative disease of unknown origin. Higher incidences for ALS in certain population subgroups have raised questions about feasible causes and led to controverse discussions, though without providing a real explanation for over 90% of sporadic ALS cases so far. Although speculative to this date however, a combined view on recent data obtained in different scientific fields might offer reasonable explanations for the origin of ALS. Here it is discussed, whether enhanced activation of the sympathetic nervous system (SNS) in concert with raised uncoupling of the mitochondrial respiratory chain in muscle tissue might be a potential key factor that could initiate the onset of ALS. The aim of this document is to show a possible link between mitochondrial uncoupling and the degeneration of motor neurons, primarily to evoke further discussions in this important area.

The first observation that tentatively connect certain activity of muscles to ALS has been published in 2003, and describes upregulation of uncoupling proteins (UCPs) in muscle tissues of ALS models (1). Briefly, certain UCPs function in muscles and in brown adipose tissues, mainly to regulate body energy expenditure (2). In general, uncoupling of the mitochondrial respiratory chain regulated by UCPs induces benefits for a single cell as well as the whole organism, e.g., to avoid or counteract stressful cellular conditions or to work against loss of body temperature in cold environment. One might guess, that UCP upregulation in muscles of ALS models might somehow be part of a protective mechanism, but without providing success. It remains unclear, whether UCP upregulation observed in the above mentioned study finally leads to an enhanced uncoupling that is capable to damage motor neurons. On the one hand, depleted ATP levels in muscles could be interpreted as a consequence of enhanced uncoupling of the respiratory chain (1). Also, numerous studies have demonstrated that UCP2 overexpression diminishes ROS production, thus could indeed function as an uncoupler (3). On the other hand, increased UCP expression without stimulator of its activity may not affect membrane potential under normal conditions sufficient to induce uncoupling. However, a muscle restricted mitochondrial defect, i.e., uncoupling of mitochondria, is sufficient to generate motor neuron degeneration (4). Furthermore, UCP upregulation has been demonstrated in invertebrate ALS models, suggesting that enhanced UCP activity and ALS could be circumspectly connected (5).

Now once believed, that under certain conditions upregulation of UCPs in muscle tissue might be a cause for ALS, rather than a consequence. This is not so far-fetched as uncoupling of muscle mitochondria triggers distal degeneration of motor neurons, which are the main targets for degeneration in ALS (4). In light of this hypothesis, the finding of UCP upregulation in ALS models is interesting, because UCPs are activated in muscle tissues by the SNS (6). Additionally, hyperactivity of the SNS is capable to induce muscle wasting, one of the initial symptoms of ALS (7). So may it possible to draw a feasible connection of mitochondrial uncoupling in muscles, activity of the SNS and ALS?

In the event muscle tissue actively participates in origin of ALS, a direct correlation of muscle mass to ALS incidence must exist. In fact it has been described in a number of studies, that ALS sex ratio prefers men and in addition, sex ratio may also change with age. In young patients the sex ratio is 2:5, however drops down in older patients to 1:4 (8). If compared to women, men in general do have more skeletal muscle mass (9). Furthermore, men may experience greater loss of total muscle mass with age than women, supporting the idea that incidence of ALS might be triggered by or depends on, thus is directly connected to muscle mass (10). However and not only because of the unknown etiology of ALS, direct involvement of muscle tissue in ALS is unclear. Also contributing to the fact that ALS sex ratio prefers men could be based upon possible protective hormonal factors in women or an increased likelihood of males being exposed to putative risk factors (11). As the case may be, no seizable explanation for ALS origin derived from these discussions has been isolated so far.

Notwithstanding, these data allow the hypothesis, that under certain conditions upregulation of UCPs in muscle tissue might be a cause for ALS, and the SNS actively participates in this process. If it should be so, more evidence must exist that connects ALS incidence to mitochondrial uncoupling and/or activation of the SNS. In fact, multiple studies in the past recognized that the SNS displays an enhanced activity in patients suffering from ALS (9–11). Briefly in this context, the SNS regulates acute stress response, also termed fight-or-flight action. On the physiological level, adrenalin and noradrenalin are major components of the stress response mediated by the SNS to primarily modulate the activity of tissues which express adrenergic receptors, e.g., blood vessels, muscles, or brown adipose tissues. It has to be mentioned, that the findings of modified SNS activity in ALS patients may base upon slightly sensitive assays. Thus, more detailed studies in ALS models are definitely necessary to fully understand the connection of SNS activity in ALS.

Naturally, the SNS requires a stressful event for activation, preparing the body to fight-or-flight actions. But it is also well known, that physical exercise massively activates the SNS (12). Additionally, several studies have shown that UCP expression increased in response to exercise or after exhaustive training (13, 14). Not surprisingly in this context, it has already been observed, that physical exercise is somehow connected to ALS, since repeated studies describe a higher incidence for ALS in people performing sports (15–18). Moreover, Chio and Mora summarize that ALS patients had always been slim or varsity athletes more likely than controls (19). Most strikingly, motor behavior like fight-or-flight action and also physical exercise directly involves motor neurons, which are the main pathological targets in ALS (20). Hence, again a direct correlation between the SNS, UCP, and ALS might exist.

More evidences for the contribution of UCPs to ALS derive from the following issues. People performing sports may suffer from injuries of their muscles more often than others, while muscular injuries are commonly treated and rehabilitated with electrotherapy. Besides, electrical muscle stimulation is used for targeted gain of muscle mass, which is facilitated by, e.g., football players and is also widely used by bodybuilders, and both groups display higher ALS incidence (16, 21). In line with this context, it has already been suggested, that electricity somehow could be connected to ALS and in effect, low frequency stimulation on rat skeletal muscle induces UCP expression (22, 23). These results are in line with the here presented hypothesis, that enhancement of the UCP system in muscle tissue possibly increases ALS incidence. Certainly, difficulties in finding a common parameter of either physical activity and behavior of people performing sports exist. Hence, it remains to be solved whether SNS activity in physical exercise is in fact the main factor responsible for the raised ALS incidence in these subgroups.

A higher probability of electrotherapy due to muscle injury or gain of muscle mass in athletes could only be one explanation to the raised ALS incidence in these specific groups. However, activation of UCPs in muscle tissues can be achieved by additional mechanisms, which are rather used by athletes or bodybuilders than the rest of the population. On the one hand, for bodybuilders loss of fat is an important matter to shape the body. For this purpose, it is not uncommon that bodybuilders use so-called uncouplers, e.g., Dinitrophenol which function likewise to UCPs (24, 25). On the other hand, administration of beta-2 agonists also induce UCPs by mimicking SNS activation, and can be used to improve muscular function (26). As already stated above and again, a higher incidence for ALS in these subgroups could be connected to UCPs, which lowers the probability of being coincidence.

In summary, overexpression of UCPs without obvious intrinsic requirement for a fight-or-flight response in muscle tissue might be a potential cause for the onset of ALS and probably also explains, why in diverse studies people performing sports or using so-called uncouplers are found to be more often affected by ALS than others. Furthermore, ALS sex ratio may be based upon by the higher muscle mass of men compared to women. Also as stated above, men may experience greater loss of total muscle mass with age than women, thus provide explanations why the sex ratio changes with age. In this respect, a preliminary study describes treatment of ALS patients with clenbuterol, an agonist of beta-adrenoceptors. In healthy patients, activation of beta-adrenoceptor induces muscle growth, however in ALS patients only fasciculations, enhanced nervousness, or cramps can be observed without any positive influence on muscle wasting (27). Concerning to the here presented hypothesis, because in ALS the SNS is already hyperactivated by UCPs and induce muscle wasting, enhanced nervousness could be judged as a sign of even more enhanced activity of the SNS. Thus, treatment of ALS patients with beta-agonist enhances the symptoms, rather to diminish it, which is in line with the outcome of the above stated study and the here presented hypothesis.

Detailed discussion of proteins contributing to stress response pathways is beyond the scope of this discussion paper, remarkably however, the picture that is emerging from the here presented hypothesis points toward a pivotal role for UCP activity in overall stress response pathways. Thereby, specific stress response mutants, e.g., superoxide-dismutases, already used as models for ALS are associated with rare familiar ALS forms (28). In this regard, it has been described recently that mutations in the gene encoding for Valosin-containing protein (VCP) contribute to 1–2% of familiar ALS cases, and strikingly, lead to enhanced mitochondrial uncoupling (29). VCP participates in specific cellular stress response pathways which have recently described to be directly linked to mitochondrial function (30–32).

Given a potential source, treatment capabilities of ALS arise in the form of, e.g., genipin, a geniposide that is present in high amounts in gardenia fruit extracts which is used as a traditional herbal medicine in Asia for centuries (33, 34). Genipin has been shown to inhibit UCP2 expression *in vivo*. Likewise in diabetes treatment, genipin could be used to diminish symptoms, because enhanced UCP expression has been demonstrated to inhibit release of insulin from beta cells (35). Here I believe it is no coincidence, that both altered sympathetic function and insulin resistance have been observed in ALS (36). Although clinically dominated by neuromuscular disturbance, ALS brings about unambiguous signs of altered sympathetic function that become clear when studied in detail (37). Consequently, if under ALS conditions the UCP activating threshold is much lower due to SNS hyperactivity, treatment with UCP inhibitors could interfere with the feedback circuit that maintain high UCP expression and in turn induces SNS activity. Further, a combined therapy using antisympathetic drugs or beta-adrenoceptor antagonists might induce benefits to slow down progression of ALS symptoms by interfering with the same feedback loop stopping adrenergenic receptor signaling. In this context, sympathetic block has already been used as a therapy of spasticity in multiple sclerosis, which is also a symptom in ALS (38).

Further research on UCP signaling especially in muscle tissues may provide more detailed insights and would help to describe the functional interplay between organs, mediated by the SNS, and which role distinct mitochondrial processes, for instance autophagy, play in ALS. Above all, more studies on SNS activation by UCPs and *vice versa* are necessary to better understand the mechanisms that might be responsible for the origin of ALS.
